# Modelling stem cell differentiation related processes—A practical overview for biologists

**DOI:** 10.1002/1873-3468.70395

**Published:** 2026-07-01

**Authors:** Ricco Zeegelaar, Xinqi Yan, Lucas F. Jansen Klomp, Stefano Schivo, Janine N. Post

**Affiliations:** ^1^ Quantitative Biology Lab, Department of BioEngineering Technologies University of Twente Enschede the Netherlands; ^2^ Department of Applied Mathematics University of Twente Enschede the Netherlands; ^3^ Department of Computer Science Open University Utrecht the Netherlands

**Keywords:** abstract model, computational modelling, deep learning, gene regulatory networks, multiscale model, ODE model, stem cell differentiation

## Abstract

Stem cell differentiation is central to development and regenerative medicine, but the complex underlying processes hinder our ability to control it experimentally. Computational models can help form hypotheses and generate predictions. There are many types of computational models available to aid in understanding stem cell differentiation–related processes, but for an experimental biologist, it might be hard to select the modelling approach that matches their research question, and to know what data would be needed to make use of the model. This review is aimed at experimental biologists to introduce various modelling types, relate them to the types of questions these models can help answer and outline the necessary data to gain new insights. The review discusses mechanistic dynamic models, both ordinary differential equation (ODE) and abstract, multiscale models and data‐driven deep learning approaches. Each model class is introduced with what the model represents, the insights it can provide, validation strategies and limitations. With this review, we want to make it easier to incorporate modelling within experimental workflows for stem cell differentiation–related research, to aid experimentation and accelerate discovery.

## Graphical abstract

Stem cell differentiation is complex and difficult to control experimentally. This review introduces suitable computational modelling approaches that can support stem cell research, from mechanistic ODE and abstract models to multiscale and deep learning methods. It explains what each approach can reveal, what data are needed and how the models can guide experiments.

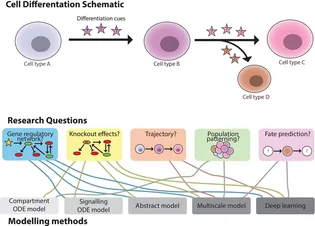

## Abbreviations

ABM, Agent‐based model

ANIMO, Analysis of Networks with Interactive Modelling

CPM, cellular Potts model

CRISPR, clustered regularly interspaced short palindromic repeats

DL, deep learning

FCS, fluorescence correlation spectroscopy

FRAP, fluorescence recovery after photobleaching

FRET, Förster resonance energy transfer

GRN, gene regulatory network

IHH, Indian hedgehog

iPSC, induced pluripotent stem cell

KO, knockout

ODE, ordinary differential equation

PDE, partial differential equation

PKN, prior knowledge network

qPCR, quantitative polymerase chain reaction

RNA‐seq, RNA sequencing

scATAC‐seq, single‐cell assay for transposase‐accessible chromatin using sequencing

scRNA‐seq, single‐cell RNA sequencing

SHH, Sonic hedgehog

TF, transcription factor

VAE, variational autoencoder

WNT, Wingless/Integrated

β‐Cat, β‐catenin

The use of stem cells provides the potential for the development of novel treatments for a wide range of diseases. For example, stem cells can be used to study early tissue formation, stem cell‐derived organoids can be used as models for testing novel therapies or for understanding the studied biological system, and stem cell‐derived cells can be used directly to replace damaged tissue. Using stem cell‐derived cells and tissues is promising, but the decision‐making processes behind cell lineage and tissue formation are complex and therefore hard to control, reducing the practical applicability of the cells. To deal with complexity, researchers use abstraction and computational modelling to test hypotheses on differentiation. Here, the use of computational modelling as a support for stem cell research is discussed.

The complexity of biological systems often means that experimental approaches are insufficient to truly understand them. A classic broken radio analogy illustrates this well; experiments may reveal crucial components that determine whether a radio will function, but this does not directly explain how interactions between components produce the system‐level behaviour required to repair a broken radio [1]. To solve this in stem cell‐related research, computational modelling serves as a tool to understand and reveal (dynamic) molecular mechanisms beyond what can be directly inferred from experimental observations alone. The goal of modelling is not to replace wet‐lab experiments, as modelling alone cannot establish that a hypothesis is true [2]. The models essentially help conceptualise biological processes and translate complex hypotheses into experimentally testable predictions. Additionally, mathematical models help interpret quantitative experimental data, as quantitative hypotheses are needed to link measured variables, such as timing, location and concentration to the underlying biological processes [2].

Models are typically constructed in the context of an iterative modelling cycle, where experimental results are used as input to construct the model, and the outputs of the computational model aid the understanding of the experiments or lead to novel ideas for experiments [3, 4]. The drastic increase in the amount of cellular data, like single‐cell and spatial datasets, has enabled modelling approaches to account for heterogeneity within cell populations. Please note that, as the number of parameters (Box 1) in a model grows, model risks such as overfitting or parameter nonidentifiability also increase. The key to preventing these modelling problems is to match model complexity to the data quality and quantity. The goal of a model is not to maximise details, but rather to maximise insights while minimising assumptions.

Box 1 **Common terminology in modelling biological networks**

**Node:** A variable that represents a biological entity in a model or network (like a gene, protein or cell state).

**Edge:** A relationship between nodes that defines the influence (like activating and transition).

**Topology:** The structure of the network, which contains all nodes and edges.

**Parameters:** Numerical values defined at the start of the simulation, which will determine the model behaviour. This refers to settings you adjust before running the model; the state of a node during the simulation is not a parameter.

**Fitting:** Adjusting parameters so that simulations reproduce experimental data.

**Simulation:** Letting the model run over time to observe outcome under the starting conditions of the model.

**Perturbation:** Altering a specific node in the model to test the influence on the simulation outcome. For example, observe what happens during a knockout of a node by removing incoming and outgoing edges.

**Validation:** Testing the model's validity using model predictions. This is done with data not used in the construction or fitting process.

The aim of this review was to guide cell biologists in how to understand and use computational models. The review is centred around decisions to make for the modelling process, rather than providing an extensive overview of modelling tools. We answer the following question for stem cell differentiation research: given the research question and available data, what class of computational model is useful and what do you need to use it? The perspective in this review is shaped by our work at the interface of experimental biology and computational modelling, where we study molecular mechanisms of tissue development and disease to construct digital twins of cartilage and heart systems. We therefore discuss how different modelling approaches can be applied to questions in stem cell differentiation.

## The modelling process

### Modelling is driven by research questions and available data

In the context of cell differentiation, computational models can assist in predicting cell fate under perturbations, reconstruct lineage trajectories from data, explain mechanisms behind differentiation and predict tissue organisation during differentiation, etc. (Box 2). The choice of a modelling tool is dependent on the question that the biologist wants to answer. However, the properties of the data used to construct the model heavily determine what type of model can be built (Box 3). Therefore, after formulating a biological research question and considering what tools are available, it is necessary to generate data with properties appropriate for the modelling tool.

Box 2 **Categories of research questions addressed in the modelling of stem cell differentiation research.**

**Table:** 

Question type	Example
Mechanistic explanation	Which molecular components and regulatory interactions explain lineage specification?
Intervention/modulation	How will changing a specific component or condition, such as a gene knockout, affect cell fate?
Dynamic trajectories (Cell state transitions over time)	How do cells progress from one fate to another?
Population‐level patterning	How do organoids and tissues self‐organise?
Predictive mapping (Data‐driven predictions)	Can I predict cell fate from omics data?

[Correction added on 7 July 2026, after first online publication: Colour shading has been reinstated in the box. The shading was inadvertently omitted from the previous version.]

Box 3 **Properties of data.**

**Table:** 

Category	Detail		Example
Depth	Sparse	Dense	Gene expression: Few markers vs genome‐wide
Time	Snapshot	Time‐resolved	Tissue images: one time point vs time‐series/tracking
Resolution	Bulk	Single‐cell	Sequencing: bulk vs single‐cell RNA‐seq
Perturbations	Absent	Present	Differentiation: untreated vs inhibitors/agonists
Spatial context	Absent	Present	Tissue cell types: isolated cells vs spatial transcriptomics

### Validating a model

Each modelling approach has its own challenge or risk, and some general pitfalls (Box 4). The models therefore need to be validated, which should be planned from the start. Generally, some data must explicitly not be used when constructing a model so that the model can later be validated using that independent data. Alternatively, the model can be validated by testing known effects that were not used as input in the model, or by directly doing a wet‐lab experiment to test predictions of the model. Commonly, a model might not pass the validation. This gives valuable insights into missing information, parameters that were not fitted correctly, or essential parts of the system that are not yet fully understood. These insights allow for the creation of an improved model, which in turn can be validated again. This iterative cycle of model improvement is common across many modelling modalities and crucial for gaining the best insights from the modelled system. The following real‐world case study illustrates this process.

Box 4 **Common risks in modelling.**

**Overfitting:** The model is fit on noise or artefacts of a dataset. It will therefore excellently replicate the data, but will fail with predictions on independent datasets.

**Unidentifiable parameters:** Multiple parameter sets can reproduce the same data, so parameters do not have unique values.

**Poorly interpretable model components:** A model can reproduce the observed data while relying on parameters or including quantities that do not correspond to measurable biological quantities, therefore limiting biological interpretation.

**Modeller bias:** Prior expectations of the modeller influence the model design which influences the outcomes.

**Wrong topology:** Key interactions or nodes are missing, so the model will reproduce data with an invalid mechanism.

#### Case study

Researchers experimentally observed that embryonic stem cells adapt to activin A signalling over time, showing differences in downstream SMAD4 induction despite continued exposure. They hypothesised that this adaptation could be due to depletion of the activated receptors of activin A, or that there might be a feedback mechanism affecting SMAD4 signalling. They made two signalling‐based ordinary differential equation (ODE) models for the different hypotheses. The receptor degradation model could explain the adaptation, but only under conditions where the recovery time of the adaptation was significantly slower than the adaptation itself. Experimentally, however, the researchers could show that the recovery time of the SMAD4 response was similar to the adaptation time. The feedback models could reproduce the dynamics of activin A adaptation. The combination of experiments and modelling therefore helped the researchers identify which of the biological mechanisms best matched their system (Based on: [5]).

## Computational modelling of cell differentiation approaches

A few approaches to model various aspects of cell differentiation are highlighted. These include mechanistic models, multiscale models and deep learning‐based approaches. Each modelling approach is described following the same questions:

- When should one use this approach?
- What do you get out of the model?
- What does the model biologically represent?
- What data do you need for such a model?
- What are the limitations of such a model?

Additionally, an application for every modelling approach is shown using a case study. In this case study, a researcher is differentiating iPSCs towards mesodermal cells using the signalling compound Wingless/Integrated (WNT). The researcher uses the different modelling classes based on the research question to answer. The models presented in the case study are hypothetical, with interactions based on networks in open‐access databases [6].

### Mechanistic models for differentiation

A modelling approach that is particularly relevant for modelling time‐dependent processes is the use of ODEs. In the context of predicting differentiation outcomes, ODEs are mainly utilised in two ways: compartment‐based models and signalling‐network‐based models.

#### Compartment‐based ODE models: Modelling differentiation as state transitions

In compartment‐based models, differentiation is explained through discrete switching between cell types. Each compartment typically describes the number of cells in a certain cell state, for example stem cells, progenitor cells or differentiated cells. In the model, the transition between different compartments is described without including detailed information on the intracellular mechanisms that lead to these transitions.

##### When to use

Compartment‐based models are useful when you want to understand which cell states occur during differentiation and how quickly cells move between the states over time, at the level of a cell population. The models explain how quickly cells proliferate, die or commit to a lineage. They can also be used to evaluate how a perturbation (e.g. physical constraints, cell number, nutrients and morphogens) will influence cell fate decisions or what the key transitions are behind the lineage decisions.

##### Insights from the model

Compartment‐based models can be used to answer biological questions at the cell population level. For example, after fitting the transition, proliferation and decay rates, *in silico* experiments on population dynamics can be performed [7, 8]. Through parameter sensitivity analysis, it is possible to identify ‘critical’ components of the differentiation process where small changes in conditions significantly change the outcome [7, 8, 9]. Moreover, it is possible to compare model performance for different hypothetical lineage structures to identify likely differentiation paths or essential mechanisms for differentiation [10, 11, 12]. These insights can, for example, be used to optimise a protocol by identifying which specific cell transitions and which rate parameters play the strongest role in the final cell‐type yield.

##### Mathematical abstraction

Compartments can be a cell type, thus a biologically defined cell state, such as pluripotent stem cell, progenitor cell or differentiated cell. Cells switch between states with certain probabilities or rates. Mathematically, the model tracks how many cells are in each state over time and does not describe the molecular details of each individual cell. This high level of abstraction to describe cells makes the model easy to construct, as the model contains fewer parameters and variables than models that aim to describe more detailed mechanistic processes underlying differentiation. Typically, the rate of change of each compartment depends directly on an inherent decay rate and the number of cells in the previous cell state. Prior knowledge can be used to identify dependencies of these rates on the context of the modelled system, for example with feedback from differentiated cells [13, 14], specific intracellular factors [11, 15], external influences [13] or a small signalling subnetwork in the larger compartment‐based model [16].

##### Data needed for the model

To determine the decay rates and transition rates for compartment‐based models, data can be used from flow cytometry, time‐resolved population measurements or lineage proportions under various perturbations [15, 17, 18]. In other work, multiparameter immunophenotyping is mentioned as a technique that can be used to track both cell fate and number of divisions (proliferation) over time [10]. From these types of data, parameters such as proliferation rate or probability of differentiation can be directly estimated from the experimental measurements of population growth and transition frequencies. Numerical estimation of parameters based on an optimisation procedure is also possible if no direct measurements on transition or decay rates are available [8, 13, 19].

##### Limitations

Compartment‐based models only represent data at the cell population level and therefore cannot be used directly to determine which genes or intracellular processes underlie such critical components of differentiation. Additionally, despite having fewer parameters than models that describe intracellular signalling, parameter identifiability issues are still common for compartment‐based models. In general, it is likely that parameter choices are not unique when fitting a model to data that describes the number of cells in different populations. This should be considered when interpreting the results obtained from a compartment‐based model.

##### Validation and risk mitigation strategies

For general validation of compartment‐based models, predicted compartment sizes can be compared with measurements from flow cytometry, immunostaining or other techniques that can quantify cell populations over time. It should also be evaluated whether the model can predict population dynamics beyond the data used for fitting. This can be done by validating the model predictions on independent datasets, perturbation conditions or different starting conditions. Validation can also be performed by explicitly splitting the data into a training, test and validation set [11]. Testing alternative compartment structures can further help reduce the risk that the model reproduces population dynamics for the wrong biological reasons.

##### Case study

A researcher, who has been using WNT to differentiate iPSCs towards mesodermal cells, forgot to add WNT signals during her differentiation and suddenly saw an unknown cell type appearing that she never saw when adding WNT. She had multiple hypotheses, one of which was that this could be a reversible cell type that dedifferentiates easily. To investigate whether this hypothesis could explain her lab results, she makes two compartment‐based models (Fig. 1). She chooses the parameters of her models to match her experimental data. She assumes that the rate of differentiation and dedifferentiation of the unknown cell type is the same. She also assumes that under WNT signalling, cells are 10x more likely to differentiate towards mesodermal cells compared with the unknown cell type. Without WNT signalling, the iPSCs never become mesodermal cells. The results of her simulations match her observations in the lab: the unknown cell type only appears at the end of her differentiation when there is no WNT signalling, confirming the validity of her hypothesis. An additional application of the model can now be to modify the parameters to see which parameter has the biggest effect on the population outcomes and change her differentiation protocol accordingly.

**Fig. 1:**
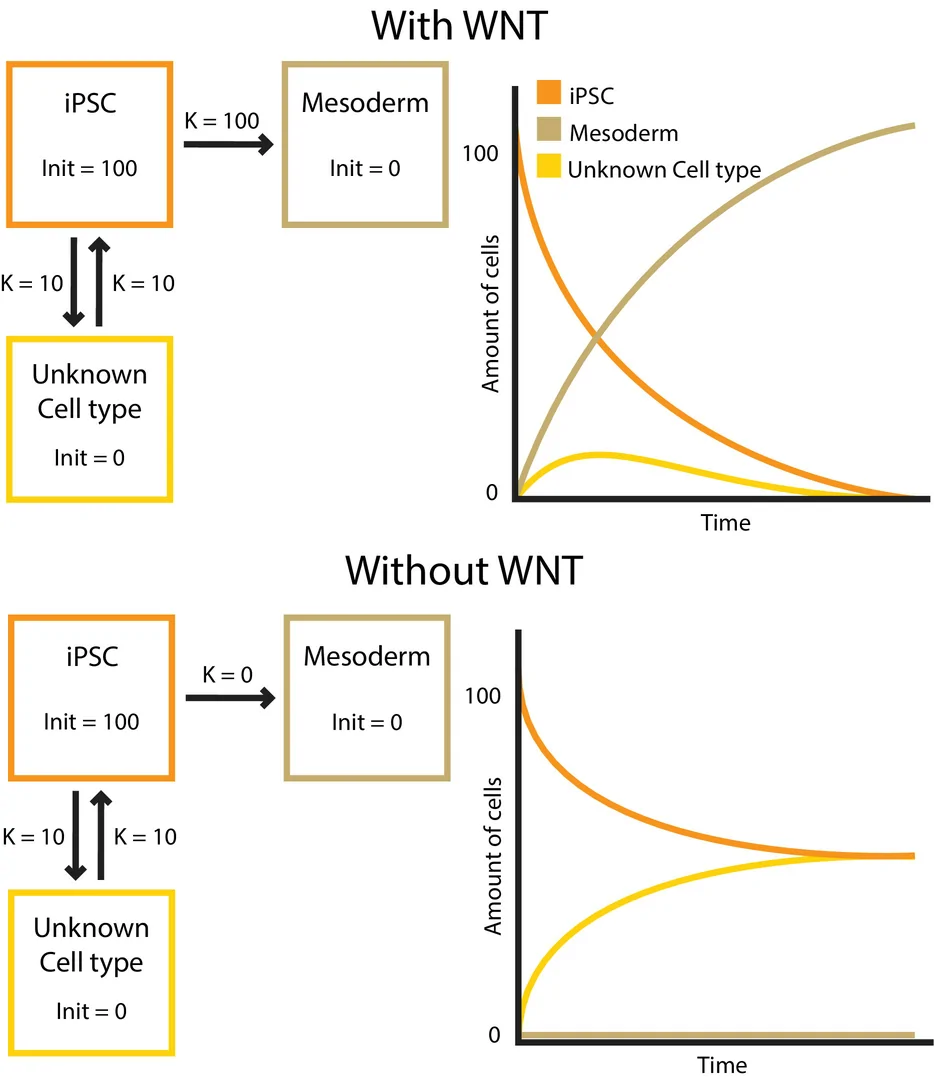
Hypothetical compartment‐based model of induced pluripotent stem cell (iPSC) to mesoderm differentiation with and without Wingless/Integrated (WNT) signalling. On the left is the model topology, on the right are the simulation results. With WNT signalling, all iPSCs eventually become mesodermal, and the unknown cell type never appears. However, without WNT signals, culture will also contain the unknown cell type.

#### Signalling‐network‐based ODE models: Modelling intracellular logic

In signalling‐network‐based ODE models, genes and proteins are explicitly represented and connections between these genes and proteins dictate how gene expression and protein activity change over time.

##### When to use

Signalling‐network models are best suited for questions about causal intracellular decision logic and signalling crosstalk in a dynamic system of a single cell. This single cell can be assumed to be representative for a population of the same cell type.

##### Insights from the model

ODE models based on intracellular networks can be used to answer detailed questions about the mechanistic processes underlying cell fate decisions. For example, these models have been used for predicting effects of perturbations [20], identifying influential nodes [21] and determining the importance of specific interactions using parameter sensitivity analysis or bifurcation analysis [22, 23]. These insights can be readily translated to an experimental setting, for example through the suggestion of a specific knockdown or overexpression experiment. Models based on signalling networks can also be used to test and validate current hypotheses in stem cell biology, with unexpected behaviour aiding the formulation of new hypotheses [24].

##### Mathematical abstraction

The model is based on a signalling network which includes various complex interactions between proteins and genes. Specifically, nodes and edges can represent a ligand binding to its receptors, an intracellular signalling pathway, activation of specific transcription factors or expression of and interactions with target genes.

##### Data needed for the model

To make signalling‐based ODE models, you need (A) the topology of the model containing all the interactions and (B) the strengths and dynamics of the interactions. For determining the dynamics of the signalling‐network‐based models, one needs dynamic readouts, that is readouts on how the system evolves over time. Examples of data types include time‐course gene expression (bulk or single cell), protein abundance and phosphorylation over time, reporter assays, and/or RNA velocity‐derived kinetics. In addition, data from perturbation experiments can be used to verify regulatory interactions and therefore aid in the development of the topology. Examples of this are knockout and overexpression data, chemical inhibition or stimulation and CRISPR–based perturbations. For these types of models, one does not need large‐scale data sets or dense time series [4, 25]. The topology of a signalling‐based ODE model can be inferred using prior knowledge or using novel experimental data.

##### Prior knowledge networks

For signalling‐network models, time‐dependent intracellular data on activity (as opposed to presence or expression) is used. In most cases, the network structure or topology is built based on prior knowledge, leading to so‐called prior knowledge networks (PKN). This network structure determines interactions or which protein/gene influences another protein/gene. All literature sources and databases describing these interactions can be used.

Online network databases, including STRING, NetworKIN, PhosphoSitePlus, BioModels and KEGG, can assist in network identification [26]. The exact reaction rates do not need to be precisely known to make a working model, as they can be inferred from molecular biology (e.g. western blot, qPCR and RNA‐seq) and biophysical techniques (e.g. FRAP, FRET and FCS) [4, 27, 28, 29, 30, 31].

##### Data‐driven networks

If time‐resolved (single‐cell) RNA‐seq or multi‐omics data is available, a data‐driven model can be generated. Importantly, cellular signalling is rarely fully captured by a single omics modality. RNA‐seq can provide detailed information on downstream transcriptional responses, but does not directly measure post‐translational events, such as protein activity or localisation, which can be equally important for the signalling dynamics. There is no gold standard for constructing data‐driven networks for mechanistic modelling, though the use of multi‐omics‐based methods is particularly promising as these methods typically lead to connections that are more mechanistically feasible [32, 33, 34]. In some cases, network construction can be simplified by modelling clusters of genes rather than individual genes [35, 36] or by focusing only on a functional network ‘motif’ that recapitulates the key dynamics [37, 38].

Thus, for inferring the topology of a signalling‐based ODE model, if a reasonably complete regulatory network is known, a PKN based model allows for mechanistic hypothesis testing with limited data. If the network structure is less well described, or if novel interactions are necessary to describe the underlying biological process, data‐driven models can be used to learn regulatory interactions from omics data.

##### Limitations

A downside of using intracellular network‐based models, be it PKN or data‐driven network models, is that there is no golden standard for constructing the network. Prior knowledge networks require detailed information on which genes and proteins are relevant, as well as information on the relevant interactions between these genes and proteins. The interpretation of the relevant interactions is subjective and therefore subject to modelling bias. At the same time, data‐driven networks can be unreliable when the data is noisy, incomplete or when the inferred interactions are wrong. Another concern when building signalling‐network‐based models is the rapid increase of the number of parameters in the model as the number of modelled genes grows. This makes parameter unidentifiability issues common for this modelling method, and therefore proper assessment of robustness is essential [20, 39].

##### Validation and risk mitigation strategies

For signalling‐based models, both the model topology and parameters should be validated. To validate the topology, alternative topologies should be tested where possible to reduce the risk of modelling bias. To validate parameters, sensitivity analyses can be performed to evaluate which interactions and parameters strongly influence the model predictions, and it should be evaluated on a case‐by‐case basis whether such highly influential parameters are due to biological effects or limitations in the model construction. Model parameters can furthermore be evaluated whether simulations of the model are able to predict dynamics under independent conditions, of gene expression at timepoints not used and of results from perturbation experiments. Predicted effects of knockdown, overexpression or pathway inhibition can be directly compared with the experimental outcomes. These experiments can further enhance the confidence that the model uses the relevant signalling dynamics.

##### Case study

A researcher wants to add WNT to his iPSC culture without the cells differentiating towards a mesodermal cell type. Inevitably however, when he adds WNT to his iPSC culture, he sees an increase in mesodermal markers (TBXT and MESP1) and a decrease in IPSC markers (NANOG and OCT4). To understand how to prevent this from happening, he needs to understand the mechanisms of this up‐ and downregulation, so he decides to make a signalling‐based ODE model (Fig. 2). He bases his ODE topology on prior knowledge from public databases and infers the parameters of interactions from how fast he sees the expression decrease and increase in his experiments. When he runs the model with different concentrations of WNT, the simulation results match what he saw in his experiment. Based on this model, he identifies that β‐catenin (β‐Cat) is a good target for a knockout, to prevent the upregulation of the mesodermal genes and the downregulation of the iPSC gene when adding WNT. With his model, he can now perform *in silico* knockout, and indeed sees that now he can add WNT to his culture without the iPSCs differentiating.

**Fig. 2:**
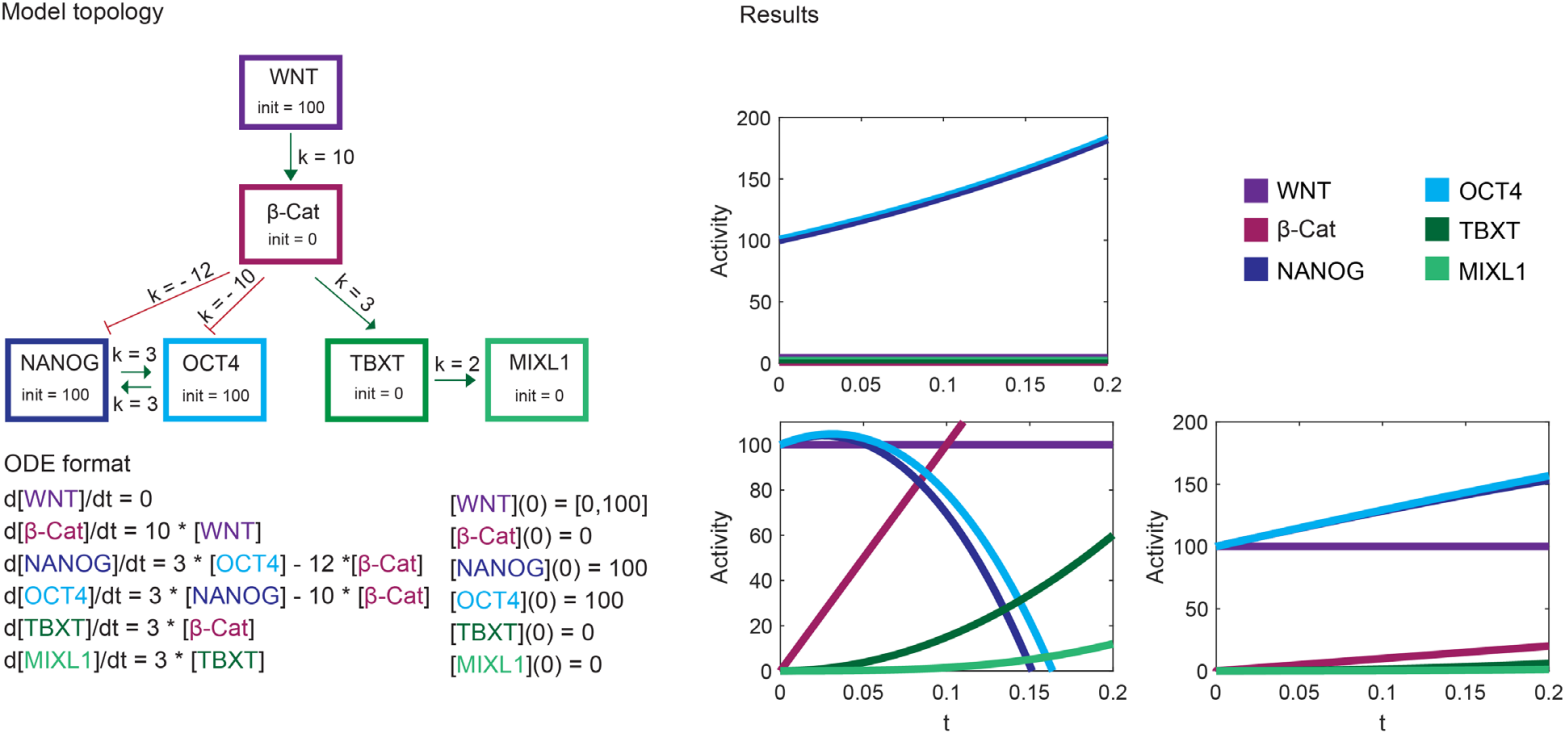
Example signalling‐based ordinary differential equation (ODE) model of the Wingless/Integrated (WNT)‐induced differentiation of induced pluripotent stem cells (iPSCs) to mesodermal cells. On the left is the model topology in schematic and ODE format. On the right are the simulations under varying initial WNT activities and β‐Cat knockout. The model predicts that WNT signalling causes the iPSCs to differentiate towards mesoderm, but β‐Cat knockout can prevent this.

#### Abstract (semi‐quantitative) mechanistic modelling

Abstract or semi‐quantitative mechanistic models are models that describe how biological components influence each other, without requiring exact reaction rates. They often use simplified activity levels, logical rules and qualitative reaction rates. This allows for obtaining important mechanistic insight while relying less heavily on lab data when building the model.

##### When to use

The context of application is the same as for the ODE models, with the distinction that this type of model allows for parameters to be uncertain or missing or inferred from semi‐quantitative time‐series data. The relative timing between events is still preserved, as is the concept of causality.

##### Insights from the model

Abstract mechanistic models can provide several types of insights in the differentiation process and are intuitively constructed in programmes, such as ANIMO [4, 40, 41]. Their mechanistic causal structure and temporal order of signalling events allow one to explain how different signalling pathways influence important transcription factors [3, 42]. Thanks to the ability to describe time‐dependent behaviour, this type of mechanistic model allows a researcher to distinguish between transient, sustained or delayed pathway activations and explain the effect of network motifs, such as feedback loops on the differentiation process [4]. Moreover, systematic perturbation‐based analyses (knockout or activation of groups of nodes) can be applied to the models to identify key players in the network dynamics [42].

All these *in silico* experiments together contribute to the main task of mechanistic abstract models, which are hypothesis generation and experiment prioritisation [3, 4, 31]. This is particularly useful when considering combinatorial hypotheses, where multiple interventions are explored at the same time: this type of experiment is notoriously difficult to conceive, and this is therefore also where the support of modelling is most useful.

##### Mathematical abstraction

Mechanistic models that do not use ODEs still include information about reciprocal influences among biological entities, often abstracting the concept of biochemical concentration to discrete levels. For example, a gene can be represented as a ‘switch’ which can be either on or off (a.k.a. Boolean variable, hence Boolean networks); more generally, a discrete number of levels is used to represent the currently active (e.g. for a kinase, a phosphorylated state, as opposed to a nonphosphorylated, inactive state) fraction of the molecular population. Examples of this type of models include Petri nets [30], fuzzy logic models [43] and ANIMO models [44].

Another typical abstraction involves the definition of reaction kinetics: instead of using numerical values related to biochemical reactions as is done in ODEs, more abstract models use a qualitative approach, or provide the choice among a few quantitative alternatives. For example, in a Boolean network, an interaction is defined as a simple formula that directly determines the activity of a downstream node, such as ‘node B is active if node A is active and node C is inactive’. In ANIMO, interactions can be assigned a qualitative speed (‘very slow’, ‘slow’, ‘medium’, ‘fast’, ‘very fast’), where protein production would be considered much slower than post‐translational modification. While this unlinks a model from a direct correspondence with real‐time events, the sequential order of events is still preserved.

The discussed model abstractions are often based on approximations of actual biochemical reactions. For example, the model underlying ANIMO is effectively a discrete‐level approximation of an ODE model [41]. This discrete abstraction allows the model analysis to be efficient, and it additionally enables the possibility to query models for specific properties through the technique of model checking [3, 45]. Beyond just simulating a network's behaviour, model checking allows it to be used to answer questions, such as ‘under which conditions is it possible to reach this wanted situation?’, ‘does the model ever reach a state in which X happens?’ and ‘is it true that whenever situation X occurs, then situation Y is always observed later on?’

##### Data needed for the model

The type of data needed to build a non‐ODE mechanistic model is in general the same as the data needed for an ODE model. The main difference is that this more abstract type of model is less dependent on precise values for parameters. This type of model is therefore more viable in cases of limited availability of experimental data, provided that the data still include information from a time series of at least three time points [4].

##### Examples of the model

Abstract modelling has been implemented for multiple processes where parameters are unknown and hard to directly infer. A Boolean model approach has, for example, been used to identify how sequential gene regulatory networks orchestrate the formation of heart field‐specific cardiomyocytes [46]. Key genes or pathways in diseases can also be identified using this type of model, as showcased by a timed automata‐based computational model of the signalling network of articular chondrocytes [3].

##### Limitations

Being more abstract than ODEs, this type of model is also less precise: it is therefore unrealistic to expect a close match between a time‐series graph obtained from a mechanistic model simulation and a corresponding time series derived from a lab experiment. Still, qualitative comparisons about the general behaviour can be made and are a useful guide for hypothesis generation and experiment prioritisation.

##### Validation and risk mitigation strategies

Being more qualitative compared with the ODE approaches, the validation of abstract models is focussed on the qualitative agreement with the biological reality. Model evaluation can therefore focus on testing the correct responses to pathway activation, order of activation and final model outcomes. Here, outcomes of perturbations such as knockouts and overexpression are also useful for comparison with experiments or literature‐reported observations. As the logical rules of the model are often simplified compared with reality, the robustness of these rules is best tested by varying the initial states and reaction rules.

##### Case study

Another researcher faces a similar problem to the previous case study: he wants to add WNT to his iPSC culture, without the cells differentiating. When he adds WNT to his iPSC culture, however, he sees an increase in mesodermal markers (TBXT and MESP1) and a decrease in iPSC markers (NANOG and OCT4). He uses the same approach as with the signalling‐based ODE to infer the topology, but he does not have experimental data to infer the parameters of the interaction. He therefore uses his biological intuition to infer the parameters, that is which processes are fast (molecules interacting) and slow (transcription and translation). When he runs the model, the simulations match the outcomes of the ODE‐based approach, which also shows that β‐catenin (b‐Cat) is a good target for knockout (Fig. 3).

**Fig. 3:**
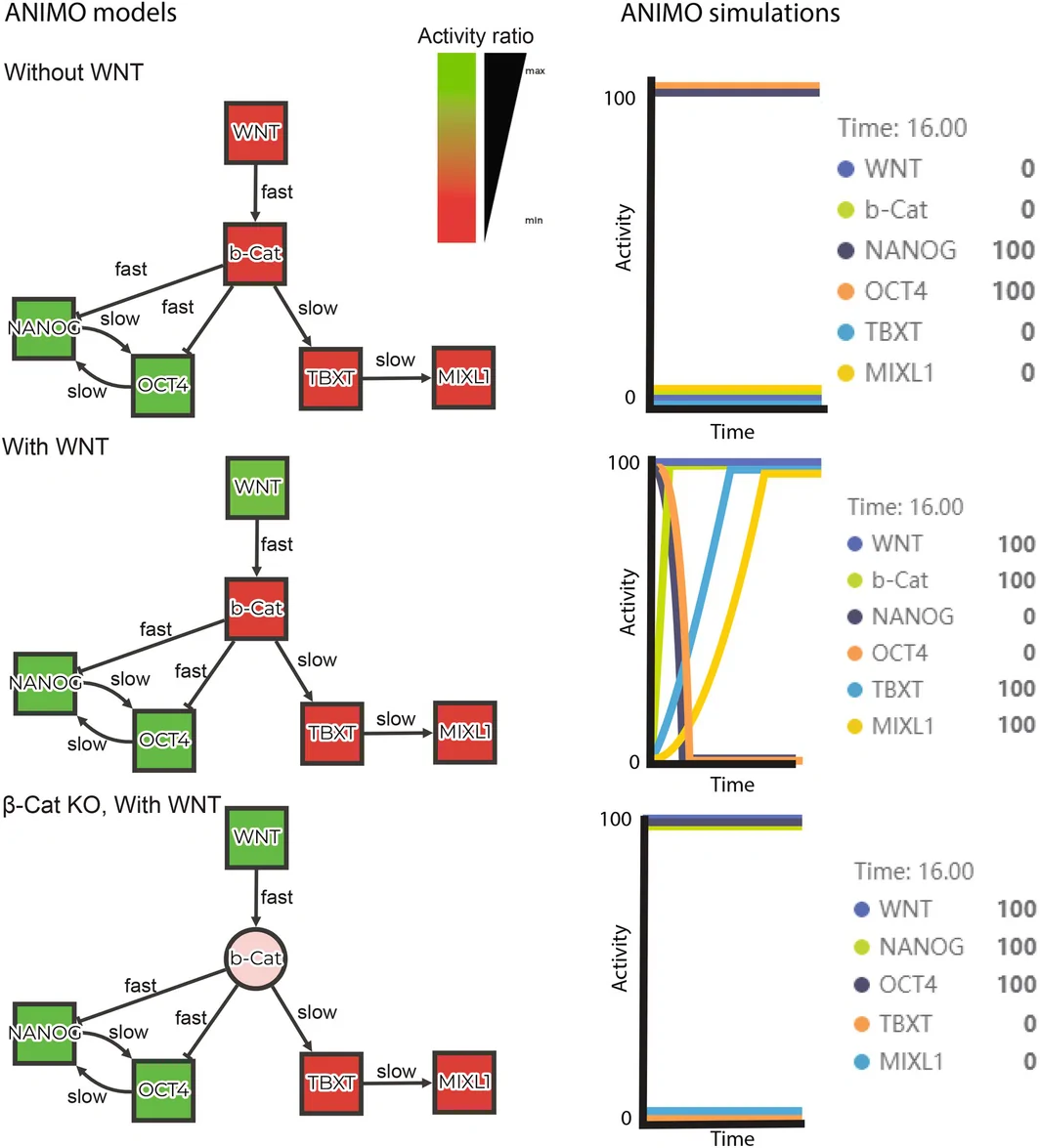
Hypothetical abstract mechanistic model of Wingless/Integrated (WNT)‐mediated induced pluripotent stem cell (iPSC) to mesoderm differentiation using modelling tool Analysis of Networks with Interactive Modelling (ANIMO). On the left, the ANIMO models are shown. Note that here no exact reaction rates need to be defined for the model to work, abstract definitions like ‘fast’ and ‘slow’ are sufficient. On the right is the simulated activity of the nodes. The model predicts that WNT signalling causes the iPSCs to differentiate towards mesoderm, but β‐Cat knockout can prevent this.

### Multiscale models: When space and cell–cell interactions matter

Stem cell differentiation in a tissue can be influenced by the spatial context of the cell. Here, differentiation and tissue organisation outcomes change not only due to intracellular interactions, but also due to cell–cell interactions, forces on the cell and extracellular signalling gradients. Although these processes affect each other, they operate on different scales, which highlights the need for a model that combines these scales. As these models can be built using many different formalisms, this section's focus is on the purpose of linking scales in stem cell differentiation, the types of scales that are regularly connected and the data needed for such models, rather than providing a complete overview of all multiscale modelling methodologies.

#### When to use

Multiscale models can be used when the research question depends on processes that interact across different spatial, temporal or functional scales [47]. They can connect intracellular regulation to cell behaviour, cell–cell organisation and tissue‐level phenomena [7]. This enables testing hypotheses and processes that cannot be easily tested with even the most cutting‐edge wet‐lab experiment. For example, a researcher wanting to link tissue organisation with other cellular processes could answer questions, such as ‘how will tissue organisation change as a result of a gene knockout?’ and ‘with what seeding density can I expect the most of a cell type with my differentiation protocol?’ A researcher more interested in spatial signalling could answer research questions like: ‘how far do morphogens penetrate a tissue?’, ‘how will tissue penetration affect the tissue size and density?’ and ‘what is the effect of adding a signal locally instead of globally?’ These questions require different modelling approaches that are normally modelled separately, but multiscale modelling allows for their integration.

#### Insights from the model

Multiscale modelling can link the effects of processes, such as tissue organisation, spatial signalling, gene knockouts, pathway perturbations, differentiation, signalling protocols, cell interactions, mechanics and forces on a tissue. Because the different scales of the systems are linked explicitly, variables in one scale can influence factors in other scales. When factors are coupled to another scale, their output can be quantified there as well. For example, if the intracellular ODE is assigned to individual cells in the tissue scale, the variables of the ODE can be tracked over time per cell. This makes it possible to link perturbations at one scale to long‐term effects at another scale (e.g. inhibiting a signalling pathway for 4 h and assessing how that will change spatial organisation after 10 days). The scales to include in the models will therefore also vary based on the question to be answered [48].

#### Mathematical abstraction

‘Multiscale model’ is an umbrella term for all models that include more than one scale to answer a research question. In the context of cell differentiation, four general scales can be distinguished. In the **subcellular** scale, there are biological processes, including protein interactions, protein localisation, protein turnover and signal transduction. The **cell** scale involves changes in cell shape, polarisation, movement, division and apoptosis. The **mesoscale** includes cell–cell interactions and multicellular actomyosin cables. Finally, on the **tissue** scale, there are morphogen gradients, mechanical inputs and processes like convergent extension and tissue size control [7]. How to mathematically model the processes within a scale largely depends on the nature of the question, as previously reviewed [2, 7, 47, 48].

#### Data needed for the model

Multiscale models often originate from linking models in singular scales together. Many modelling formalisms exist for describing the same scale in different ways, and therefore there is no established universal dataset for constructing such a model. For example, at the cellular scale, in some systems cell behaviour is driven by chemotaxis and differential adhesion, while other systems are mainly driven by tension and pressure. Data needed to describe the system will therefore largely be determined by the properties of the system. The approaches to get from data to a multiscale model have previously been reviewed [7, 48].

In the context of cell differentiation, the ability of multiscale models to provide tissue organisational insights from interactions in other scales is highly valued. For the tissue scale, the rules driving the organisation during stem cell differentiation can be derived from basic images, live imaging and force inference techniques, or even spatiotemporal information from multiple single‐cell transcriptomic datasets [22, 49]. Here, the effects of mechanical, signalling or geometric perturbations are important for deriving these rules.

#### Examples of the model

Due to the large number of systems that can be described using multiscale modelling, applications vary a lot. For example, one multiscale model was used to prove the relevance of dedifferentiation plasticity for long‐term cancer stem cell population maintenance, revealing a new target for cancer treatment [50]. Another group used multiscale modelling to provide proof of the proposed mechanisms behind experimental outcomes. They disputed a fundamental paradigm in liver regeneration, showing that SHH rather than IHH was the best way to represent hepatocellular activity [51].

#### Limitations

Because multiscale models combine processes at different biological scales, the limitations are tied to the modelling choices made at each scale. With the large number of parameters that are often included in a multiscale model, acceptable parameter sets are most of the time not accurate to the biological reality. Making a multiscale model is labour intensive and requires different types of data. Errors at the single‐scale level can produce seemingly correct multiscale behaviour for the wrong reasons. Because multiscale modelling covers a wide variety of model types and combinations of scales, there is no universal validation strategy that applies to all multiscale models. The dependence on model‐specific assumptions, parameter choices and validation decisions therefore makes these models sensitive to modeller bias [7].

#### Validation and risk mitigation strategies

Validation of multiscale models should be done in layers, and specific to the model. The single‐scale models must be individually validated relevant to that scale before validating at the multiscale level [48]. The validation of intracellular components can follow the strategies described for signalling‐network models, whereas the spatial and tissue‐scale components can often be validated using live imaging data, spatial transcriptomics, tissue size, morphology or marker localisation patterns. After passing single‐scale validation, the multiscale model should be compared with system‐level data that was not used to build the model. For example, a multiscale model of a tissue can be compared with spatial organisation, tissue size changes and cell‐type distributions. Perturbation experiments are also useful here, as they test whether the model can capture results across scales, rather than just reproducing the original condition. Sensitivity analysis and testing alternative parameter sets can additionally help define whether model conclusions are robust or whether the model applications should be restricted to generation of hypotheses.

#### Case study

A researcher noticed that when using an iPSC to mesoderm differentiation protocol on a 3D spheroid of iPSCs, the outer layer of cells differentiated faster than the inner cell layers. She hypothesised that this might be because the WNT compound she is using has a limited penetration inside the tissue, rather than fast differentiating cells just migrating to the outside. To investigate whether this hypothesis could be true, she made a multiscale model of the differentiation (Fig. 4). She used the ODE previously defined in the case study for the signalling‐based ODE. For the cell properties, she made sure that they were the same between iPSCs and mesodermal cells so that there could be no other properties like differential adhesion that might influence the layering in the tissue. To mimic the 3D situation, she modelled that WNT signals were only on the outside of the spheroid and could slowly diffuse inwards. She combined the extracellular, intercellular and intracellular rules in a model and ran a simulation. She observed that the cells on the outside of the spheroid were expressing TBXT earlier than the cells in the middle, supporting the hypothesis that limited WNT penetration could explain faster differentiation in the outer layer.

**Fig. 4:**
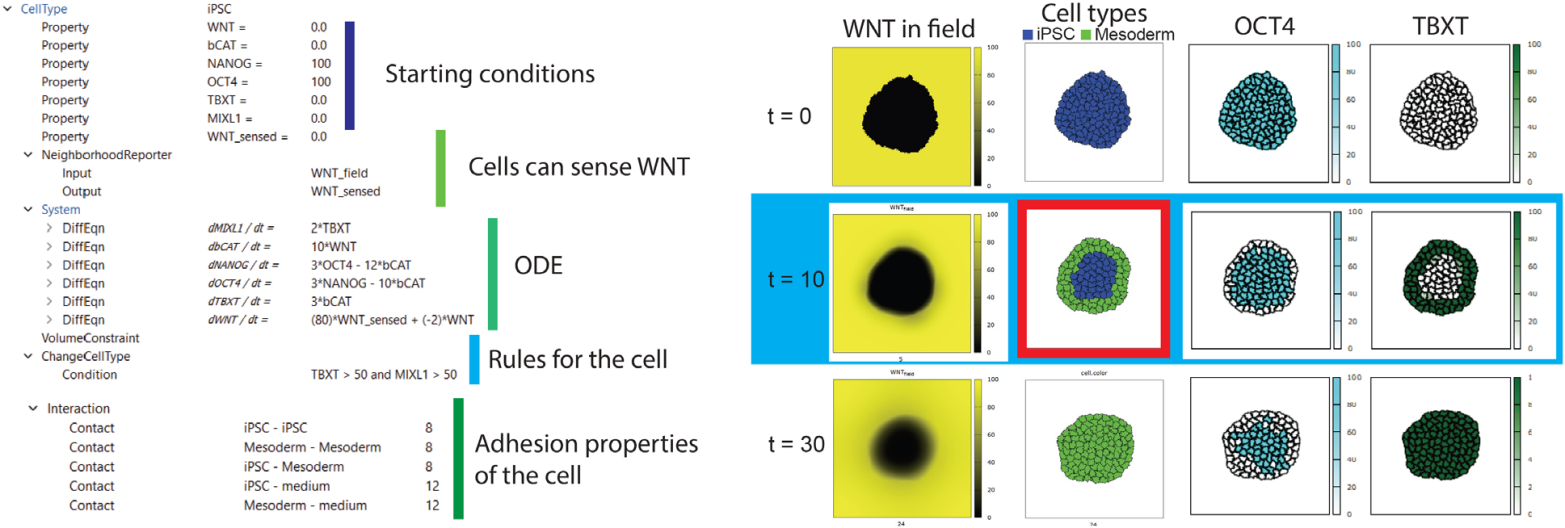
Two‐dimensional multiscale model of Wingless/Integrated (WNT)‐mediated induced pluripotent stem cell (iPSC) to mesoderm differentiation in a 3D spheroid. On the left are the rules for the different scales within the multiscale model. On the right, snapshots of the multiscale model are shown. At the timepoint t = 10, the outer layers of cells seem to have differentiated into mesodermal cells, while the inner layer of cells still has an iPSC cell identity.

### Deep learning and data‐driven dynamical modelling

Recent advances in single‐cell omics have caused a rapid increase in data resolution and amount of data available, but mechanistic details cannot automatically be inferred or measured. Deep learning (DL) has been gaining popularity as a tool to get various insights from this complex data, such as trajectory reconstruction of cell lineages, fate prediction of differentiating cells and inferring the regulatory dynamics behind cell fate. When modelling stem cell differentiation, it is important to predefine the biological research question and find appropriate deep learning tools to apply.

#### When to use

Deep learning approaches are useful to consider when the research question involves intrinsically complex large‐scale data. Importantly, insights from DL rely on the full dataset rather than needing to abstract away any measured genes. DL can be used to bridge different modalities of data to gain higher levels of biological insights. For example, it can combine scRNA‐seq (transcriptome) with scATAC‐seq (chromatin accessibility) or spatial data for more robust downstream tasks, such as differential expression analysis or cell‐type annotation [52]. Deep learning approaches can also be used to determine the dynamics of a system. With time‐series data of a differentiation experiment, instead of just determining the static states that the cell lineage followed by preselecting specific genes or their interactions, DL can help predict how cell states evolve over time and progress towards a specific cell fate [53]. Additionally, DL methods can predict how a cell reacts to a perturbation (e.g. a drug or knockout) or elucidate which combinations of cues (cytokines, small molecules and genetic perturbations) push cells towards desired fates before doing expensive experiments.

#### Insights

Similar to using ODE‐based models, DL‐based dynamical models can be used as ‘virtual differentiation experiments’ if you design them with interpretable outputs [53, 54]. These models of cell differentiation can reconstruct the continuous trajectory of differentiation from discrete ‘snapshots’ (cells), revealing transition states and lineage branching that exist only briefly between stable cell types [55]. In contrast to mechanistic ODE models, which often require an explicit PKN structure with selected variables, DL methods are not restricted by the analysis to predefined gene sets or curated interactions. In addition, while ODE‐based models formalise specific mechanistic hypotheses, deep learning models allow for hypothesis flexibility, allowing regulatory patterns to emerge from the data without committing to a preset structure. For example, for cell differentiation trajectories, dynamic DL models can estimate the direction and speed of change, predict future cell states [56], propose causal drivers of differentiation rather than just correlative markers and identify how the ‘neighbourhood’ (e.g. proximity to a niche cell) instructs a stem cell's fate.

#### Mathematical abstraction

To infer differentiation lineages, some DL models [e.g. variational autoencoders (VAE) and diffusion models] treat differentiation as the movement of single cells in a learned low‐dimensional state space. This space captures the developmental trajectories of the cells in the data. The distances between the cells within this space therefore are assumed to reflect developmental progression. A DL model predicts how the cell state changes from one to the next [53]. These models can then help infer the most probable developmental lineage of cells under perturbations, by simulating how a cell would move through the learned space.

#### Data needed for the model

The main bottleneck in utilising DL for cell differentiation is not the DL architecture but having the right data with sufficient quality and coverage of the dynamics. To generate a DL model that captures the full lineage trajectories during cellular differentiation, the following data is required.

To learn specific differentiation dynamics, multiple time‐resolved scRNA‐seq measurements across the differentiation process are highly advantageous, ideally from early, mid and late stages (e.g. Days 0, 3, 6 and 9), covering the entire differentiation process. These data should come from consistent protocols and metadata (time, dose, medium, passage, line and batch) so that the model can disentangle biology from technical noise. Denser temporal sampling, for example every 6–12 h, particularly around critical fate transitions, can substantially improve the models' capability to resolve rapid regulatory changes and transient intermediate stages. The model can additionally be improved by pairing the scRNA‐seq data with scATAC‐seq data or phospho‐protein measurements from the same cell, to discourage the model from inferring influences that are mechanistically implausible [32].

#### Deep learning tools

Methods like scVI, which is an example of VAEs, learn a continuous latent representation of single‐cell transcriptomics data, and when combined with trajectory analysis tools, such as Monocle3 [57], facilitate the identification of rare cell state transitions and support downstream trajectory inference [58]. Using PRESCIENT, a neural ODE‐based method, it is additionally possible to learn continuous differentiation dynamics and predict the temporal evolution of cell populations [59]. Variational autoencoder‐based models, such as scGen, have been used to predict transcriptional responses to gene perturbation by knockout or drug treatment, enabling *in silico* prioritisation of experimental intervention [60].

#### Limitations

The biggest hurdle in using deep learning‐based models to infer dynamic processes in stem cell differentiation is the interpretability. Deep learning methods do not automatically become mechanistically plausible, even when you add data modalities. Current deep learning methods do not truly generate dynamic models that capture temporal changes in (signalling) networks. The deep learning methods focus primarily on static network inference from cross‐sectional omics data. A model might predict cell fate with 99% accuracy but fail to tell you which genes or why certain genes led to that decision (the ‘black box’ problem). As the full reasons behind the outcomes of deep learning methods cannot be interpreted, validation is critical. It is, however, challenging to define a ‘ground truth’ on single‐cell dynamics or lineage trajectories, making it hard to define the accuracy of predictions.

Overfitting is another common problem for DL, where if the data is too sparse, the model may ‘fill in the gaps’ with patterns that look plausible mathematically but are biologically false. This eventually leads to generalisation problems, where models trained on one dataset will completely fail on a different dataset. Because deep learning is a very active field, researchers are currently working on tools that counter these limitations.

#### Validation and risk mitigation strategies

Deep learning models are typically validated by splitting the dataset into a training, validating and testing dataset. Next to good performance on unseen data points, it would also be useful for validating deep learning‐based models to consider how well models generalise by evaluating the performance on data that lies outside the distribution of data found in the training data. Additionally, predictions can be validated against wet‐lab experiments. For example, Squidiff, a generative tool that predicts transcriptomics profile shifts between cellular states, was validated by predicting cellular response to a drug combination experiment and comparing the predictions against experimental measurements using Pearson correlation coefficients [55].

#### Case study

A researcher wants to derive the lineages that cells follow during her 2‐day iPSC to mesoderm differentiation protocol. For the cell states within the lineages, she is also curious what the GRNs look like. She therefore isolates 500 cells from day 0 of her protocol and 500 cells from day 2 of her protocol and performs scRNA‐seq on every single cell. Using a deep learning trajectory analysis for the cells, she can see what lineages the cells followed (Fig. 5). Surprisingly, besides the expected lineage from iPSC to mesoderm, some cells also differentiated towards an unknown cell type. She inferred the cell types using differential gene expression analysis, and wants to know how the genes regulate each other in the cell types. Because she does not possess the prior knowledge on the GRN in the cell types, she used a deep learning‐based GRN inference tool on all the clusters from her trajectory analysis. With this, she now knows the key regulators for the cell states that appear with her protocol, and she also has an indication of what the off‐target cell type might have been. With the insights of the deep learning tools, she can optimise her protocol to prevent cells from differentiation towards the off‐target cell type.

**Fig. 5:**
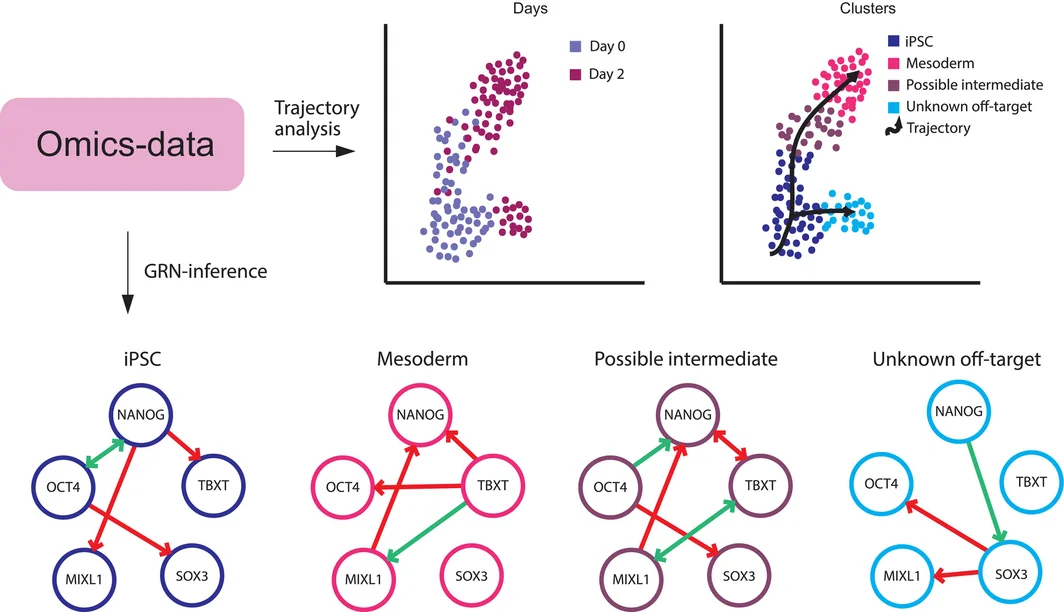
Trajectory analysis and gene regulatory network (GRN) inference using single‐cell transcriptomics data from cells isolated from induced pluripotent stem cells (iPSCs) to mesoderm differentiation. The trajectory analysis revealed four clusters: the iPSC state, mesoderm state, an intermediate state between iPSC and mesoderm, as well as an off‐target state. Using the GRN inference, the key regulatory genes and their interactions could be determined, revealing more about the cell types and possible targets (green = positive influence on expression, red = negative influence on expression).

## Choosing the modelling approach

Choosing the modelling approach should be based on the type of research question and available or obtainable data. As a starting point, Table 1 serves as a flowchart to summarise the modelling approaches per research question. For a type of research question (Box 2), the table shows what should be modelled, what modelling method(s) are available, and tools available for the modelling type (Table 2). For example: with the goal of figuring out population‐level kinetics, lineage proportions over time are needed to make a compartment‐based ODE model. With the goal of figuring out intercellular causal logic, when the regulatory topology is available or can be assembled, a signalling‐network‐based ODE model can be used. If the spatial context of the cells is dynamic and needs to be considered for the differentiation outcomes, multiscale models are recommended. For trajectory reconstruction or fate prediction from high‐dimensional time‐resolved datasets, deep learning‐based models and approaches are available.

**Table 1:** Common research questions for stem cell differentiation research, available modelling strategies and possible tools to use for modelling.

Mechanistic explanation		What to model	Model family	Method	Tools
Through what mechanism do	Signals influence expression?	Signalling + GRN	Dynamic model	Signalling‐based ODE/abstract	T1, T2, T3
	Signals influence lineage?	Signalling + GRN + lineages	Dynamic model	Signalling‐based ODE/abstract	T1, T2, T3
			Deep learning	VAE‐based generative model	T11
	Signals influence tissue organisation in a developing tissue?	Extracellular + intercellular + cell	Multiscale model	PDE (extra) + ODE (inter) + CPM (cell)	T4
				ABM (extra + cell) + ODE (inter)	T2 + T5
	Signals influence tissue organisation in a developed tissue?	Extracellular + cell	Multiscale model	PDE (extra) + CPM (cell)	T4
				ABM (extra + cell) + ODE (inter)	T2 + T5
	KO/genetic modifications influence lineage?	GRN + lineages	Dynamic model	Signalling‐based ODE/abstract	T1, T2, T3
		Expression + lineage markers	Deep learning	Transformers/attention	T12
	KO/genetic modifications influence tissue organisation in a developing tissue?	Extracellular + intercellular + cell	Multiscale model	PDE (extra) + ODE (inter) + CPM (cell)	T4
				ABM (extra + cell) + ODE (inter)	T2 + T5
	KO/genetic modifications influence tissue organisation in a developed tissue?	Intercellular + cell	Multiscale model	ODE (intercellular) + CPM (cell)	T4
				ABM (cell) + ODE (inter)	T2 + T5

Interventions/modulations		What to model	Model family	Method	Tools
How do perturbations	Of a pathway change cell fate	Probabilities/rates + signalling + lineages	Transition model	Compartment‐based ODE	T1
		Signalling network + lineages	Dynamic model	Signalling‐based ODE/abstract	T1, T2, T3
	Of a pathway change expression	Signalling network + GRN	Dynamic model	Signalling‐based ODE/abstract	T1, T2, T3
	Of a gene change tissue composition	Probabilities/rates	Transition model	Compartment‐based ODE	T1
	Of a gene change expression	GRN	Dynamic model	Signalling‐based ODE/abstract	T1, T2, T3
			Deep learning	Time‐resolved expression prediction	T13
				*In silico* knockout	T18

Dynamic trajectory		What to model	Model family	Method	Tools
What is the lineage of cells	During a differentiation protocol?	Single‐cell expression profiles	Deep learning	VAE/latent model + trajectory analysis	T7 + T8
				Neural ODE/fate dynamics	T9
			Machine learning	RNA velocity	T10
	In a tissue?	Spatially resolved single‐cell profiles	Deep learning	Optimal transport/dynamic matching	T14

Population‐level patterning		What to model	Model family	Method	Tools
How will tissue patterning change	In response to external forces?	Intercellular + force‐based interactions	Multiscale model	Vertex‐based model (cell)	T6
	In response to well mixed signals?	Continuous extracellular + intercellular + energy‐based cell	Multiscale model	PDE (extra) + ODE (inter) + CPM (cell)	T4
	In response to low quantity signals?	Discrete extracellular + intercellular + energy‐based cell	Multiscale model	ABM (extra + cell)	T2 + T5
	In response to gene knockout?	Multiscale model (with signalling/forces)	Multiscale model	PDE (extra) + ODE (inter) + CPM (cell)	T4
				ABM (extra + cell) + ODE (inter)	T2 + T5

Predictive mapping		What to model	Model family	Method	Tools
From omics data, can I predict	Cell fate during differentiation?	Dynamic trajectories	Deep learning	Trajectory inference	T9, T13
	Key transcription factors that orchestrate lineage decision?	TF prediction	Deep learning	TF importance scores	T15, T18
	Key markers for a cell type?	Gene expression dynamics	Deep learning	Neural ODE	T9
	The GRN at timepoints that I measured?	GRN inference	Machine learning	Static inference	T15, T16, T17

[Correction added on 7 July 2026, after first online publication: Colour shading has been reinstated in the table. The shading was inadvertently omitted from the previous version.]

**Table 2:** Tools index.

Tools		Category	Ref
Mechanistic			
T1	Matlab/Python/Julia	Programming languages commonly used for ODE modelling.	
T2	COPASI	ODE	[61]
T3	ANIMO	Timed automata	[44]
Multiscale (tissue level)			
T4	Morpheus	Cellular Potts model	[62]
T5	FLAME	Agent‐based model	[63]
T6	Chaste	Vertex/force‐based	[64]
Deep learning			
T7	scVI	Single‐cell latent/integration	[58]
T8	Monocle3	Trajectory analysis	[65]
T9	PRESCIENT	Trajectory/fate prediction	[59]
T10	scVelo	RNA velocity/dynamics	[66]
T11	scGEN	VAE‐based generative model	[60]
T12	scGPT	Transformers/attention	[67]
T13	Squidiff	Trajectory inference	[55]
T14	Waddington‐OT	Optimal transport	[68]
GRN inference			
T15	DeepRiG	Regulator prioritisation	[69]
T16	ANANSE	TF activity/enhancer networks	[34]
T17	SCENIC+	Regulons/network Inference	[32]
T18	DANSE	Dynamic Network Inference	[25]

[Correction added on 7 July 2026, after first online publication: Colour shading has been reinstated in the table. The shading was inadvertently omitted from the previous version.]

In some cases, multiple modelling approaches can be used to address the same research question, such as ODE‐ and DL‐based methods. The model to be considered depends on how much prior knowledge is available and whether mechanistic interpretability is required. ODE‐based models are preferred when known interactions need to be included in the model, for example, to test causality. In contrast, deep learning approaches are preferred when there is data available, but limited prior knowledge is available about the system, at the cost of results having reduced interpretability. Therefore, careful consideration should be given to if a hypothesis‐driven approach or a data‐driven paradigm is the most suited with the given system understanding and data availability. Having chosen a modelling approach, the next steps should include identifying which validation strategies and risk mitigation steps are most applicable for the specific use case, as discussed in the validation and risk mitigation sections.

## Conclusions and perspective

Processes regarding stem cell differentiation are complex and difficult to unravel from experiments alone. Computational models can assist in mechanistic interpretation, help in forming and testing hypotheses on these processes, and enable *in silico* experiments that complement wet‐lab work. Many modelling frameworks exist, each requiring different data, assumptions and yielding different outputs. To make modelling more accessible to experimental biologists, and to aid in the decision of which modelling method fits for the research question to be answered, several modelling approaches were highlighted using practical decision questions.

Especially now that it is becoming easier and more common to generate large amounts of data, it is increasingly important to put effort into understanding how to translate measurements into insights. All computational methods have their advantages and limitations, and understanding them is the key to making effective use of the available data. Strengthening modelling understanding among experimental biologists therefore supports the generation of the most informative data and greatly aids in effective communication with computational collaborators. With this, modelling can provide new insights that are challenging to obtain from the data alone, accelerating experimentation and discovery.

The broken radio analogy for biological discoveries [1] was made in 2002, when computational models were not a standard in the biological discovery workflow. It ended with a plea for more accessible computational methods, as well as an increase in computational literacy among experimental biologists. As reviewed, there now are many tools available that allow for discoveries beyond wet‐lab possibilities. The use of computational models has become more common, and due to the necessity to obtain insight into complex data, the computational literacy of experimental biologists has greatly improved in recent years. We believe that within the current biological landscape, there are clear opportunities for integrating modelling and wet‐lab work. Collaborations between computational and wet‐lab biologists should already start from the research question stage, up to structured validation strategies and targeted follow‐up experiments. This will reduce the need for uninformed trial and error approaches, make optimal use of datasets and speed up progress in stem cell differentiation research.

## Author contributions

RZ contributed to the conceptualisation, visualisation, writing—original draft, writing—review and editing. XY contributed to the conceptualisation, writing—review and editing. LJK contributed to the conceptualisation, visualisation, writing—review and editing. SS contributed to the conceptualisation, supervision, writing—review and editing. JNP contributed to the conceptualisation, funding acquisition, project administration, supervision, writing—review and editing.
